# Pathogenesis and Therapeutic Mechanisms in Immune Thrombocytopenia (ITP)

**DOI:** 10.3390/jcm6020016

**Published:** 2017-02-09

**Authors:** Anne Zufferey, Rick Kapur, John W. Semple

**Affiliations:** 1 Keenan Research Centre for Biomedical Science, St. Michael’s Hospital, Toronto, ON M5B 1W8, Canada; Anne.Zufferey-Bakos@crchudequebec.ulaval.ca (A.Z.); kapurri@smh.ca (R.K.); 2 The Toronto Platelet Immunobiology Group, St. Michael’s Hospital, Toronto, ON M5B 1W8, Canada; 3 Canadian Blood Services, Toronto, ON M5B 1W8, Canada; 4 Department of Pharmacology, Medicine, and Laboratory Medicine and Pathobiology, University of Toronto, Toronto, ON M5B 1W8, Canada; 5 Division of Hematology and Transfusion Medicine, Lund University, 221 84 Lund, Sweden

**Keywords:** immune thrombocytopenia (ITP), autoimmunity, T cells, B cells, platelets

## Abstract

Immune thrombocytopenia (ITP) is a complex autoimmune disease characterized by low platelet counts. The pathogenesis of ITP remains unclear although both antibody-mediated and/or T cell-mediated platelet destruction are key processes. In addition, impairment of T cells, cytokine imbalances, and the contribution of the bone marrow niche have now been recognized to be important. Treatment strategies are aimed at the restoration of platelet counts compatible with adequate hemostasis rather than achieving physiological platelet counts. The first line treatments focus on the inhibition of autoantibody production and platelet degradation, whereas second-line treatments include immunosuppressive drugs, such as Rituximab, and splenectomy. Finally, third-line treatments aim to stimulate platelet production by megakaryocytes. This review discusses the pathophysiology of ITP and how the different treatment modalities affect the pathogenic mechanisms.

## 1. Introduction

Primary immune thrombocytopenia (ITP) is an acquired immune disorder characterized by an isolated thrombocytopenia (peripheral blood platelet count <100 × 10^9^/L) [1] due to pathogenic anti-platelet autoantibodies [2,3], T cell-mediated platelet destruction [4], and impaired megakaryocyte (MK) function [5,6,7]. It can be observed in both adults and children, with both sexes being affected [8]; however, the underlying mechanisms of pediatric ITP compared to adult ITP may be different [9,10,11]. On the other hand, secondary ITP is triggered by inherited or acquired predisposing diseases such as chronic infections, including *Helicobacter pylori* and human immunodeficiency virus (HIV), or autoimmune diseases such as systemic lupus erythematosus or rheumatoid arthritis. In the infectious cases, it may be that a viral antigen is recognized as being similar to a platelet antigen, a process termed molecular mimicry, which then gives rise to cross-reactive anti-platelet autoantibodies [12,13,14,15]. Treatment of or the natural elimination of the infection eventually contributes to an increase the platelet count accompanied by a decrease in autoantibody titers [13]. Thus, these cases are usually associated with a better outcome; such is the case with most children with newly diagnosed ITP. In adults, primary ITP constitutes approximately 80% of the diagnosed patients, whereas the remaining 20% are affected by secondary ITP [16]. Primary ITP has a prevalence of up to 9.5 per 100,000 adults and an incidence of about 3.3/100,000 adults per year [17], and this increases with age [18,19]. If symptoms occur they can manifest as petechiae; purpura; mucosal bleeding in the urinary tract or in the gastrointestinal and/or oral cavities, including epistaxis [20]; and a reduced quality of life [21,22,23,24,25,26]. In the worst cases, fatal intracranial haemorrhages can occur, but this is only in about 0.2% of cases [27]. The bleeding diatheses are, however, very heterogenous, and it is still unclear why patients with similar platelet counts can present with different clinical bleeding manifestations [9].

ITP is mainly due to IgG autoantibodies, which bind to platelets and MKs [28,29,30], targeting very abundant surface antigens such as glycoprotein (GP) αIIbβ3 (GPIIbIIIA) and GPIb-IX-V [31,32]. Platelets with bound autoantibodies are subsequently recognized by phagocytes bearing Fcγ-receptors (FcγRs), which results in enhanced antibody-mediated platelet phagocytosis and destruction primarily in the spleen [2,3,33]. Moreover, autoantibody binding to MKs can inhibit their maturation or can lead to their destruction [34,35,36], and thrombopoietin (TPO), a liver derived glycoprotein hormone that drives thrombopoiesis, cannot normalize the platelet count [37]. In fact, approximately two-thirds of patients with ITP present with normal or decreased TPO plasma levels, adding a novel functional deficit of TPO to the pathophysiology of the disease [38,39,40]. In addition, autoreactive T cells are also involved in platelet [4,41] and MK destruction [42,43], and, despite an increased MK number in the bone marrow of some patients, many present signs of morphological abnormalities including apoptotic ultrastructure as well as activation of Caspase-3 [44,45]. Superimposed on these cellular impairments, the cytokine profile of patients with ITP is also imbalanced with, for example, higher serum levels of interleukin (IL)-2, interferon (IFN)-γ, and IL-17 [46,47,48].

ITP can be clinically classified into 3 phases [1] with the first phase, called newly diagnosed, occurring within the first 3 months post-diagnosis. The second phase is termed persistent ITP and refers to symptoms lasting between 3 and 12 months, and the third phase is termed chronic ITP, in which symptoms remain present beyond 12 months [1]. Acute ITP, a term originally used primarily for children, is now considered newly diagnosed. ITP is termed severe when it is characterised by the necessity of active intervention to treat bleeding symptoms. The majority of the adult patients will progress to the chronic stage [49], and several treatment modalities are now utilized, which target various aspects of ITP pathophysiology such as the inhibition of autoantibody production, the decrease of platelet destruction, the modulation of T cell activity, or the stimulation of platelet production [50]. In this review, we will give an overview of the pathological mechanisms involved in ITP and the effects of the different therapeutic regimens.

## 2. Molecular and Cellular Mechanisms of the Pathogenesis of ITP

### 2.1. B Cells and Autoantibodies

Patients with ITP produce anti-platelet IgG antibodies (and more rarely IgM or IgA antibodies) [28,29,30,51,52] which bind to platelets and mark them for phagocytic breakdown in the spleen and liver [39]. These antibodies often bind to very abundant glycoproteins on the platelet surface, particularly GPαIIbβ3 (GPIIbIIIA) and GPIb-IX-V molecules [31,32]. However, in as many as 30% to 40% of the patients, no detectible antibodies can be found [53,54]. Whether the lack of antibodies in patients is due to the robustness of the antibody tests used or perhaps due to a purely T cell-mediated mechanism is still unknown. Of interest, in those patients positive for anti-platelet antibodies, other antibody specificities beside the classic surface glycoproteins have been found, including cytosolic proteins [55], which may suggest that platelets undergo protein degradation by antigen presenting cells (APC) followed by antigen presentation to T cells [56]. Moreover, other mechanisms have been proposed to be involved in antibody production in ITP including antigenic cross-reactivity (mimicry), somatic mutation [16,53], and defects in the elimination of autoreactive B-cell clones [16]. In addition, oxidative stress, which favours the production of autoantibodies, may also be involved [57]. The type of epitope targeted by autoantibodies may also be a marker of disease severity and, to some extent, of response to treatment, in mice at least [58,59]. Indeed, it has been hypothesised that certain antibody specificities are more prone to induce platelet clearance [58] and apoptosis [60,61,62] or to inhibit megakaryopoiesis [35]. For example, anti-GPIb antibodies appear to induce a stronger platelet destruction by increasing the release of CD62P and phosphatidylserine and the clustering of GPIb receptors [63], and, in mice, these antibodies tend to be more resistant to the effects of intravenous immunoglobulin (IVIg) treatment [58]. Antibody-mediated platelet destruction has also been shown to be enhanced by the acute phase protein C-reactive protein (CRP) both in vitro and in vivo [64]. Interestingly, increased levels of CRP at the diagnosis of childhood ITP predicted a slower platelet count recovery, but after IVIg treatment, the levels of CRP dropped, accompanied by a recovery in the platelet count and decreased bleeding severity [64]. Furthermore, platelet opsonisation by autoreactive antibodies can affect platelet reactivity by modulating agonist stimulation and platelet secretory granule release [65]. This observation may partially explain the variability of ITP bleeding severity as well as the differences in response to treatment observed in some patients with similar platelet counts. Furthermore, the presence of anti-platelet autoantibodies increases the risk of thrombotic events [66,67,68], perhaps due to procoagulant microparticles released by activated platelets [69,70] or associated predispositions [37,63,71].

Autoreactive antibodies are secreted by plasma cells, which have been reported to be present at higher levels in patients with ITP [72], as well as the B cell regulator, and B cell-activating factor (BAFF, also called B cell stimulator (BlyS)), which is an important factor in B cell selection, survival, and proliferation. Indeed, BAFF promoter region polymorphisms as well as its up-regulation in the plasma have been strongly associated with ITP in humans and in a murine ITP model [73,74,75,76]. B cells were also shown to be increased in the red pulp of the spleens from patients with ITP [77] and they appear to have higher proliferative rates in these splenic areas [78]. Moreover CD19^+^CD41^hi^CD38^hi^ B-regulatory cells (Bregs), which promote peripheral tolerance, are also impaired in ITP [79,80]. They fail to reduce CD4^+^ T cell activation and trigger the recruitment of CD4^+^CD25^+^FoxP3^+^ T regulatory cells (Tregs), a subtype of CD4^+^ T cells crucial for immune suppression and tolerance [81] via IL-10 secretion [82]. The CD19^+^CD24^+^ FOXP3^+^ Breg subpopulation has also been recently shown to be significantly increased in the spleens of patients with ITP compared with control trauma patients [83]. These studies suggest that, like Tregs, the peripheral deficiency of Bregs may be due to sequestration of these cells within lymphoid compartments. Nonetheless, there appears to be a central role for Bregs and IL-10 secretion in ITP and their modulating effects on Tregs in the pathogenesis of the disorder.

Taken together, these studies demonstrate that ITP patients present with impaired plasma cells, Bregs, and B cells, leading to the production of pathogenic antibodies. These antibodies, via platelet and MK opsonisation, trigger platelet destruction in the spleen and liver as well as defective megakaryopoiesis.

### 2.2. T-Cell Imbalance in ITP

Abnormal T cells have been described in patients with ITP, including a higher T helper cell reactivity against platelets, a lower frequency of circulating CD4^+^CD25^+^FoxP3^+^ Tregs and CD4^+^ Th0, and Th1 activation patterns [33,41,46,81,84,85,86,87]. Only about 60% of patients with ITP have detectible plasma and/or platelet-bound autoantibodies [54,56], suggesting a non-antibody-mediated mechanism of ITP. Related to this, cytotoxic CD8^+^ T cells were found in the circulation of patients [4,88,89] and a similar finding was observed in an active murine model of ITP [34,87]. These CD8^+^ T cells are able to directly lyse platelets in vitro [4] and can accumulate in the bone marrow, where they are able to inhibit thrombopoiesis [43]. Furthermore, compared with healthy individuals, CD3^+^ T cells from patients with ITP have a lower rate of apoptosis and a higher clonal expansion rate, leading to abnormal cytokine secretion, including IL-2, INF-γ, and IL-10 [46], which may be responsible for the lower CD4^+^CD25^+^FoxP3^+^ Treg levels and function observed in patients with active disease [43,90,91,92,93,94,95,96,97,98,99,100,101,102,103,104,105,106,107].

Tregs, via their critical functions in maintaining self-tolerance by interacting with APC and decreasing CD19^+^ B cell and CD8^+^ T cell responses [108,109], appear to be the key cell types that may be responsible for the initiation of ITP [110,111,112]. This T cell subpopulation exists in 5% to 10% of all the circulating CD4^+^ T cells and their lower levels, and function in patients with chronic ITP [43,90,91,92,93,95,96,97,98,99,100,101,102,103,104,105,106,113,114] may reflect a dysregulated cellular immunity, leading to the autoimmune destruction of platelets. Furthermore, Tregs were shown to be significantly decreased in the spleen of thrombocytopenic ITP mice but concomitantly accumulated in the thymus [34,115]. Like Bregs, it is possible that the peripheral deficiency of Tregs is due to the sequestration of this cell type. In addition, Catani and colleagues showed that impaired communication between dendritic cells (DC) and Tregs also resulted in less tolerogenic DCs [116]. These results were supported by Daridon et al. [78], who observed a lower percentage of Tregs in the splenic proliferative lymphoid nodules in patients with ITP, confirming an impaired Treg development and suggesting that autoreactive antibody production may take place in these nodules [116]. On the other hand, Tregs are also modulated by CD16^+^ monocytes that were shown to be increased in patients with active ITP [117]. It appears that CD16^+^ monocytes release IL-12, which promotes T cell differentiation into Th1 cells and inhibits the secretion of IL-17 thereby down-modulating Tregs [118]. In addition, CD4^+^ T cell proliferation is promoted in vitro by CD16^+^ monocytes via INF-γ [117].

Tregs are not the only affected T cell population in ITP. For example, CD4^+^ Th cells have also been shown to be defective in patients with chronic ITP, resulting in increased IL-2 secretion [46] via a mechanism involving platelet GPIIbIIIa presentation and Th cell stimulation in vitro [119,120,121]. Th22 levels were also found to be elevated in patients with ITP as well as their associated cytokines, TNF-α and IL-22 [122,123,124,125,126]. In addition, other cytokines such as INF-γ have been found to be increased in adult patients with ITP [127]. Taken together, these results suggest that a Th cell defect and highly imbalanced cytokine secretion patterns exist, which may promote B cell activation [46]. Indeed, this observation is supported by several studies showing a Th1/Th2 deregulation, which may be mediated by the reduced secretion of platelet-derived chemokines and cytokines, including epidermal growth factor (EFG), chemokine C-C motif ligand 5 (CCL5), and chemokine C-X-C motif ligand 5 (CXCL5) [20,47]. Feng and co-workers showed that these chemokine/cytokine levels were associated with the severity of ITP in a passive model of ITP further supporting a crucial immunomodulatory role for platelets in ITP [47].

On the other hand, Zang et al. [99,100] and others [106,124,125,128,129,130,131] reported that Th17 cells and their associated cytokines IL-6 and TGF-β were significantly upregulated in patients with ITP, which may, in association with Treg impairment, promote a Th1-mediated immune response, triggering the disease in both humans and mice. Th17, like Tregs, differentiate from CD4^+^ T cells in the presence of IL-6 and TGF-β [132], and the secretion of IL-17 stimulates the production of pro-inflammatory cytokines such as IL-1, IL-6, and IFN-γ leading to anti-platelet antibody production in mice [131] and in patients with ITP [99,123,129,133,134,135]. This hypothesis is, however, not without controversy as others have not demonstrated a modulation of these cytokines in the plasma of patients with ITP [134].

In summary, T cells also play a crucial role in ITP. Indeed, abnormal T cell subsets, including lower Tregs and unbalanced Th17, Th0, and Th1 profiles, as well as the presence of cytotoxic CD8^+^ T cells constitute the cellular mechanisms of ITP pathogenesis.

### 2.3. Dendritic Cells in ITP

APC including DCs, macrophages and, in certain conditions, B cells, are continuously scanning their environment to process and present foreign antigens to immune cells [136,137,138,139,140,141]. In certain circumstances such as during inflammation, their function can be altered, and abnormal processing as well as enhanced self-antigen presentation can be observed, contributing to the development of autoimmune diseases. DCs are the most efficient APC [142,143], and several studies have shown their impairment in ITP [116,144,145]. For example, Catani et al. showed that DCs from patients with ITP were able to stimulate T cell proliferation upon platelet antigen presentation in vitro, probably via an increased DC CD86 expression [144]. Moreover, plasmacytoid DCs (pDCs), which are a particular subset of DCs specialized in type I interferon production (INF-α and INF-β) [146,147], are also affected in ITP. The pDCs levels were found to be lower in patients affected by either primary ITP or *H. pylori*-mediated secondary ITP [145]. It appeared that platelet counts in these patients were highly correlated with the number of circulating pDCs supporting their role in ITP pathology [145]. On the other hand, the DC enzyme indoleamine 2,3-dioxygenase 1 (IDO1) was found to be lower in patients with ITP, which would hamper the transition of CD4^+^ T cells to Tregs and therefore contribute to a Treg deficiency which may stimulate the onset of disease [116].

In addition to antigen presentation, Toll-like receptor (TLR)-mediated recognition of infectious agents has also been associated with ITP. For example, TLR4, which recognizes and binds lipopolysaccharide (LPS), a gram-negative bacterial endotoxin, and O-linked mannosyl residues of fungi, has been shown to be involved in LPS-induced ITP [74,148,149,150]. In addition, DC-associated TLR7 was also shown to induce B cell proliferation as well as anti-platelet autoantibody production in vitro via BAFF (BlyS) production [76]. Taken together, APC function, particularly in DC subsets, is abnormal and may significantly contribute to lymphocyte activation and the autoimmune pathology in ITP.

Thus, APCs, primarily DCs, are also impaired in ITP, which may suggest that abnormal self-antigen presentation takes place, which contributes to stimulating pathogenic antibody production thereby contributing to the progression of the disease.

### 2.4. Megakaryocytes in ITP

Megakaryopoiesis predominantly takes place in the bone marrow (BM) niche, a very restricted micro-environment, where specific cytokines, adhesion molecules, and growth factors regulate MK-maturation and pro-platelet release [38,151]. MKs are strongly affected in ITP, as demonstrated by impaired development (decreased ploidy and granularity) and platelet release [6]. In addition, approximately two-thirds of patients with ITP have plasma autoantibodies that are able to significantly inhibit MK maturation from TPO-treated CD34^+^ hematopoietic progenitor cells [36] and induce apoptosis [44] in vitro. It may be that different autoantibodies have different affinities for MKs and thus trigger different morphological and kinetic changes in these cells [152]. Indeed, several studies have shown that MKs were directly cleared by neutrophils and macrophages [44] despite normal or slightly elevated plasma levels of TPO [153]. Plasma TPO levels are, however, generally normal in patients with ITP compared to those with other thrombocytopenic diseases while the levels of other MK regulatory cytokines, such as IL-6 and IL-11, are increased [154,155]. The causes for these decreased TPO levels are still not clear, but TPO may be degraded along with the increased destruction of antibody-bearing platelets [153].

In ITP, MKs are clearly targeted by anti-platelet autoantibodies binding GPIb and GPIIbIIIa, and this induces both morphological and physiological changes [6,35,156]. These changes include a reduction of granules with a vacuolisation of the cytoplasm and a smoothing of the plasma membrane [6,7]. In addition, immature MKs as well as mesenchymal stem cells (MSC), which sustain MK maturation and pro-platelet formation [157], are also affected and appear to be apoptotic [44,45]. On the other hand, MKs regulate other cells in the BM niche including plasma cells, which produce antibodies [158,159,160,161], and may thus indirectly contribute to the pathophysiology of the disease. For example, the whole BM niche may be affected in ITP via MSC and MKs and their important immunomodulatory roles including the inhibition of T-cell activation and the production of IL-10 [5,162]. In patients with ITP, in addition to defective megakaryopoiesis [6,35,39,152], MSC do not appear to multiply and lose their ability to prevent CD8 T cell proliferation [157]. It seems that patients with chronic ITP present a defective vascular niche in the bone marrow, thereby reducing the interaction of MKs with the niche microenvironment, including endothelial cells [163] and plasma cells [160,161]. These studies indicate that MKs are directly affected in ITP, and the whole BM is impaired, enhancing the progression of the disease. The exact mechanisms, particularly the role of TPO, however, still remain to be unravelled.

MKs and the whole BM niche are thus also damaged in ITP. It is targeted by autoantibodies and T cells, which leads to impaired MK maturation and platelet production despite relatively normal TPO levels.

**Figure 1:**
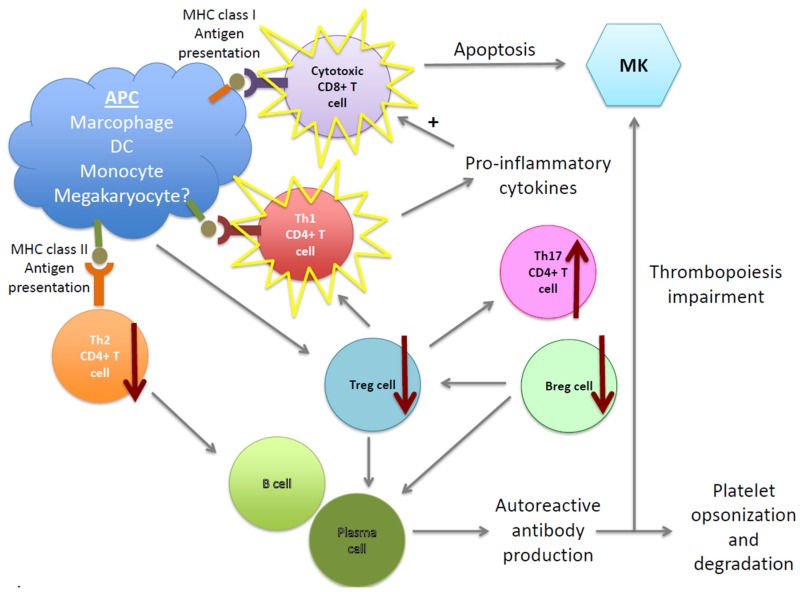
Cellular pathogenic mechanisms in immune thrombocytopenia (ITP). Multiple cells are involved in the pathogenesis of ITP. B cells and plasma cells are abnormally regulated and produce autoantibodies, which bind platelets and megakaryocytes (MKs), inducing their impairment and/or degradation in the spleen and liver. The cellular immune response is also affected, leading to a decrease of Tregs and Bregs, which contributes to autoreactive plasma cell survival (supporting autoantibody production) and unbalanced Th CD4^+^ T cell subsets. Moreover, cytotoxic CD8^+^ T cells are also activated, inducing platelet and MK apoptosis as well as the dysregulation of BM niche homeostasis. Therefore, ITP pathogenesis does not only results in platelet destruction, but also in a megakayopoiesis and thrombopoiesis defect.

Figure 1 summarizes the multiple defects associated with ITP pathogenesis including B- and plasma cell-defects as well as T cell-impairment. These abnormalities lead to both platelet and MK damage, resulting in platelet degradation and inefficient thrombopoiesis.

## 3. Therapies of ITP

Primary ITP in adults usually progresses towards chronic disease and therefore necessitates therapy aiming to restore a durable platelet count allowing sufficient hemostasis. Historically, the best way to achieve this goal was splenectomy. Indeed, 60% of the splenectomized patients presented with a normalized platelet count 5 years post-splenectomy [164,165]. Considering the potential surgical, vascular, and infectious complications, medical therapies are often considered less invasive, despite the fact that they don’t always succeed in providing long-term remission. The treatment strategies consist in stimulating platelet production to increase the platelet counts, increasing platelet half-life, and decreasing the autoreactive nature of the immune response by targeting the autoreactive antibody production and the platelet destruction.

**Figure 2:**
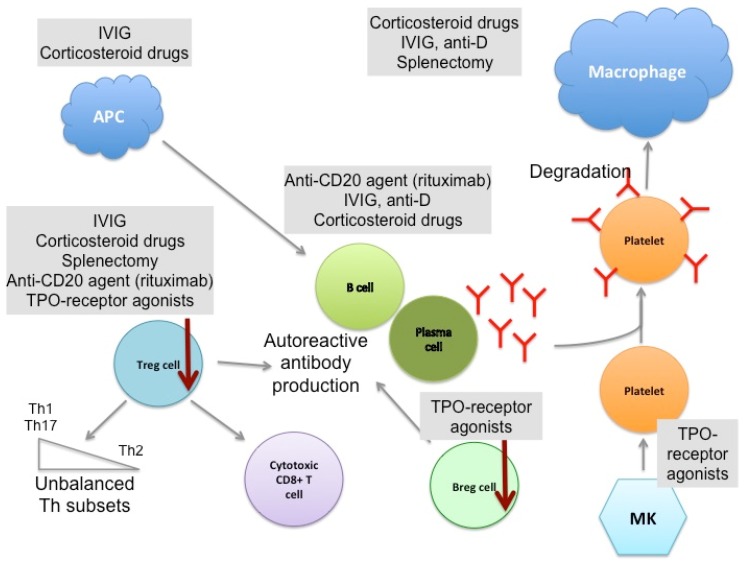
Therapeutic mechanisms of current ITP treatments. Several drugs are used to treat chronic ITP. The first line of treatment consists of corticosteroids alone or in combination with intravenous immunoglobulin (IVIg) or anti-D, which aim to decrease platelet destruction and platelet antigen presentation by antigen presenting cells (APC) to restore a normal immune response. They also act on B cells and plasma cells, thus decreasing autoantibody production, and rescue impaired Treg function. Second-line therapies include immunosupressive drugs such as Rituximab, which directly targets B cells, and splenectomy. Both treatments also modulate the T cell compartment, notably increasing Tregs. Thrombopoietin (TPO)-receptor agonists (Romiplostim and Eltrombopag), which stimulate platelet production by MKs, are third-line treatments and are used for patients who do not respond to other therapies. Here again, TPO-agonists present indirect immunomodulatory effects on Bregs and Tregs. Combining multiple therapeutic approaches is often required to ensure the restoration of a physiological platelet count.

First-line treatments include corticosteroids with or without intravenous IVIg and anti-D [166,167]. Second-line therapies consist of splenectomy and/or immune-suppressive agents such as the B cell-depleting anti-CD20 agent Rituximab, and TPO-receptor agonists such as Romiplostim and Eltrombopag are considered third-line treatments [168] (Figure 2). Recently, several studies (reviewed in [49]) have suggested that an early aggressive treatment of chronic ITP may improve the long-term outcome of patients. Indeed, the effectiveness of the treatment depends on the initial trigger of the thrombocytopenia, which is often multifactorial and targets components of adaptive immunity (T and B cells) as well as inflammatory factors (cytokines and the presence of autoantigens), as described above. Therefore, therapies targeting these two aspects of the disease may be more efficient than administrating a treatment that affects one or the other [169,170]. In addition, it is also relevant to take into account the duration of the disease in terms of response to the treatment. The chronic aspect of the disease may be the result of a B and T cell memory response and may therefore be more difficult to resolve than the primary immune response responsible for the induction of ITP. B cell clonal expansion and its positive selection may lead to a higher titer of autoreactive anti-platelet antibodies with a higher affinity for platelets and may be more difficult to combat with conventional therapies [171,172]. Further investigations are, however, still needed to better understand the transition towards chronicity and its implication for the treatment response, outcome, and cure for ITP.

### 3.1. First-Line Treatments

The primary effect of first-line treatments is to decrease autoantibody-mediated platelet clearance, presumably through Fcγ receptors [53]. Corticosteroids are pharmacological derivatives of the glucocorticoid steroid hormones, and they bind cytosolic receptors and modulate a large variety of genes, triggering many physiological changes [173]. Immunosuppressive agents (e.g., high-dose dexamethasone and low-dose prednisone together with rapamycin or rituximab) were shown in patients with chronic ITP to modulate T cells by increasing the number of peripheral Tregs, restoring the Th1/Th2 ratio, and normalizing the Th17 subpopulation consistent with an increase of IL-10 and TGF-β [90,93,174,175,176]. Immunosuppressive drugs such as prednisolone or dexamethasone also modulate B cell activation via a decrease of BAFF (BlyS) [73] and modulate DCs [96].

IVIg is used as a treatment for ITP as well as for other autoimmune diseases [177]. The working-mechanisms of IVIg are incompletely understood although several modes of action have been suggested. These include, for example, blocking antibody-mediated platelet clearance by saturating Fc receptors on macrophages; promoting the expression of inhibitory FcγRIIb via sialylated IgG Fc fragments; saturation of the neonatal FcR, which increases the clearance of autoreactive antibodies; modulation of DC maturation; and/or the modulation of T cell subsets towards a higher proportion of Tregs and a lower proportion of Th17 [177,178,179]. In addition, IVIg may also affect several other pathways such as inhibition of autoantibody production and regulation of the B cell repertoire, modulation of inflammatory cytokines such as IFN-γ [180], neutralization of autoreactive antibodies by anti-idiotype antibodies, and inhibition of the complement cascade pathway [115,178,179,181,182,183,184,185,186,187,188]. This multitude of theories on the mechanism of action of how IVIg works in ITP are somewhat enigmatic; however, the treatment is a very effective means to temporarily raise platelet counts in patients with ITP.

Patients with ITP who are RhD antigen positive and have an intact spleen can also be treated with polyclonal anti-D [189]. This treatment is prepared from the plasma of RhD negative subjects immunized against the D antigen [181]. However, like IVIg, there are more questions than answers about how this medication exactly works, and several attempts to produce monoclonal versions of anti-D have remained unsuccessful [166]. In a murine ITP model, it appeared that anti-D-coated erythrocytes competed with antibody-opsonized platelets for FcγIIIA–mediated degradation by splenic macrophages [190], and they were suggested to have a similar mode of action in patients with ITP [189,191]. It has, however, also been associated with a decrease in autoreactive antibody production in patients with chronic ITP [192], suggesting an additional effect of anti-D on B cells. Although some patients with ITP have had serious hemolytic events, this therapy, like IVIg, is very effective in temporarily raising platelet counts.

### 3.2. Second-Line Treatments

If patients with ITP fail first-line treatments or relapse, second-line treatments are necessary to manage the disease. For example, because the spleen is the primary site for platelet-reactive T and B cell activation and platelet destruction in ITP [193], it is not surprising that a splenectomy is still the gold standard for restoring physiological platelet counts in patients with ITP, and it remains the method of choice in refractory patients with ITP [49,194]. A complete remission is indeed achieved in about 60% of the patients, and another fifth of them show a partial response [164,195]. As with any surgical procedure, however, splenectomy is not without risk, and surgery-related complications have been reported in up to approximately 25% of the cases [195], including about a 1% mortality rate [165]. For example, it is well known that splenectomy is associated with an increased risk of sepsis and increased incidence of vascular complications [165]. Despite these risks, this surgical procedure is still considered the best treatment modality for the long-term increase in platelet counts in patients with ITP.

Rituximab is a chimeric antibody directed against the CD20 antigen on B cells and, upon administration, results in their virtual elimination in vivo [90,93,196,197,198,199]. It is thought to induce either B cell apoptosis or destruction in the spleen via either complement-dependant cytotoxicity or antibody-dependent cellular cytotoxicity (ADCC) [196,198,200,201]. The resulting depletion of B cells results in decreases of anti-platelet antibody titers and, interestingly, in the normalization of the T cell impairments observed in patients with chronic ITP [90,92,199] and in murine ITP [197]. These studies suggest that the mechanism of action of rituximab may in fact be an indirect regulation of the T cell compartment. However, the real benefit of this treatment remains controversial. Indeed, about 50% of resistant chronic ITP patients present a short-term response to Rituximab, and this platelet rise lasts for 6 months or more in about 30% of the cases [202,203,204,205]. Clinical variables, such as gender, duration of the disease, or co-treatment were also shown to affect the short-term efficacy of the medication [204]. In addition, recent studies have shown that only a fifth of the patients maintained a physiological platelet count 5 years post-treatment [206], and no clear long-term improvement was observed in a cohort of more than 100 patients in comparison to corticosteroid drugs [207].

### 3.3. Third-Line Treatment

Patients who fail splenectomy or Rituximab can be treated with TPO-receptor agonists. Both Eltrombopag and Romiplostim activate TPO receptors on MKs and induce platelet production via the JAK2 and STAT5 kinase pathways [208,209], and both therapies have proven efficacious in most refractory patients with ITP. In addition, it appears that approximately one third of Romiplostim-treated patients remain in remission even after 24 weeks off TPO treatment [210]. In addition to its obvious role in enhancing MK proliferation, it appears that Romiplostim can also rescue the Treg deficiency observed during active disease [91]. It was shown that Treg function was increased, and the platelet counts correlated with circulating TGF-β levels [91], which may be due the increase in the platelet mass. Similarly, Bregs were also shown to be increased in non-splenectomized patients with ITP under this therapy, concomitant with a reduction in pro-inflammatory monocytes and the enhancement of B cell immune-modulatory activity by CD16^+^ monocytes [118]. These studies suggest that TPO-receptor agonists may not only directly induce thrombopoiesis but also modulate the immune system, perhaps by modulating both Tregs and Bregs [9].

## 4. Conclusions and Perspectives

ITP is a highly complex autoimmune disease and its etiology and pathogenesis remain to be fully understood. Indeed, despite extensive genetic research pointing out the genes involved in T cell activation or the single nucleotide polymorphisms (SNP) associated with cytokines (for instance reviewed in [211]), the pre-existing factors which may promote the loss of tolerance toward platelet antigens and affect patient outcome are yet to be identified. They are multifactorial in nature and may consist of a combination of genetic predispositions, environmental conditions (including various forms of infection), and responses to treatment. ITP is characterized by autoreactive antibodies associated with impaired T and B cells, leading to the destruction of platelets and defects in thrombopoiesis and megakaryopoiesis. This pathogenic pattern is enhanced by a pro-inflammatory cytokine profile that consists of increased IFN-y, IL-2 and IL-17 as well as decreased immunosuppressive IL-10, TGF-β and IL-4, promoting autoantibody development. Underlying these defects is a central deficiency of immune tolerance due to defects in both Tregs and Bregs [212,213]. Of interest is that, although the diverse array of therapies are available for ITP that can increase platelet counts, they all seem to affect Tregs and Bregs in a similar fashion by rescuing their peripheral deficiency. Since platelets are not only hemostatic but also have significant immunomodulatory functions [20,214,215,216], perhaps increasing their counts by whatever means also sets up an anti-inflammatory milieu that alters the pathogenic immune responses seen in ITP. Thus, it is possible that although platelets are the target of autoimmune attack in ITP, they may also be able to regulate the autoimmunity against themselves. Further research will be required in order to test this interesting concept.
